# COVID-19 Vaccine Intentions Within the Medicare Population

**DOI:** 10.1093/geroni/igab046.566

**Published:** 2021-12-17

**Authors:** Tami Swenson

**Affiliations:** Des Moines University, Des Moines, Iowa, United States

## Abstract

COVID-19 vaccine intentions by older adults reflect individual care seeking behavior and medical system trust and broader systemic cultural shifts related to vaccine hesitancy. The purpose of this paper is to examine the October wave of the rapid response panel survey fielded by the Centers for Medicare and Medicaid Services (CMS) to track and monitor the effects of the pandemic within the Medicare population. With a sample size of 9686 Medicare beneficiaries, the calculated statistics use replicate weights to adjust for the complex survey sample design and balanced repeated replication using Fay’s adjustment of 0.3 for variance estimation. When asked about the likelihood of getting the COVID-19 vaccine if one were available, 58 percent of the Medicare population definitely or probably intended to get the vaccine, 16 percent expressed they would probably or definitely not, and 26 percent were not sure. Black or Hispanic Medicare beneficiaries were significantly more likely to express they would probably not or definitely not get the vaccine than White, non-Hispanic Medicare beneficiaries. Distrust of what government says about the vaccine and concern about the safety or side effects were the most common reasons for not intending to get the vaccine. Those expressing intentions to not get the COVID-19 vaccine in the October 2020 survey wave were more likely to lack access to the internet, which is a potential systematic barrier if they changed their intentions following the FDA approvals of the COVID-19 vaccines and more information became available in the winter and spring of 2021.

